# Acute kidney injury and early mortality in extremely preterm neonates born at 22–27 weeks gestation

**DOI:** 10.1007/s00467-026-07214-9

**Published:** 2026-04-10

**Authors:** Elad Resnick, Colm Travers, Russell Griffin, Namasivayam Ambalavanan, David J. Askenazi

**Affiliations:** 1 Division of Pediatric Nephrology, Department of Pediatrics, University of Alabama at Birmingham, 1600 5th Avenue South, CPPN 210, Birmingham, AL 35233 USA; 2 Division of Neonatology, Department of Pediatrics, University of Alabama at Birmingham, Birmingham, AL USA; 3 Department of Epidemiology, School of Public Health, University of Alabama at Birmingham, Birmingham, AL USA

**Keywords:** Acute kidney injury, Neonate, Extremely preterm, ELGANs, Mortality, Cohort study

## Abstract

**Background:**

Extremely low gestational age neonates (ELGANs) remain at high risk for morbidity and mortality. Acute kidney injury (AKI) is increasingly recognized, but data in the most immature neonates, especially those born at 22–23 weeks, are limited.

**Methods:**

We conducted a retrospective cohort study of inborn neonates at the University of Alabama at Birmingham between 2015 and 2021, born at 22–27 + 6 weeks gestation and ≥ 400 g without major anomalies. Early AKI was defined using KDIGO serum creatinine criteria based on postnatal days 3–7 measurements. The primary outcome was death within 7 postnatal days, and we evaluated AKI with logistic regression models to adjust for gestational age (GA), birth weight z-score, antenatal steroids, 5-min Apgar, and study year.

**Results:**

Of 813 neonates, 487 (59.9%) had sufficient creatinine data for AKI assessment. Median GA was 25.5 weeks, and birth weight was 717 g. Overall, 60 neonates (12.3%) developed AKI. Incidence decreased with advancing gestation, from 27% in 22-week GA neonates to 5% in 27-week GA neonates. Early mortality occurred in 39 of 487 (8.0%). After adjustment, AKI was associated with higher odds of early mortality (aOR 2.48, 95% CI 0.88–7.02), though this did not reach statistical significance. Severe AKI showed a stronger association (aOR 3.99, 95% CI 0.94–16.9).

**Conclusions:**

AKI incidence was inversely associated with GA in this large cohort enriched with 22–23-week neonates. Severe AKI may increase early mortality risk, underscoring the need for systematic kidney monitoring in the most immature neonates.

**Graphical Abstract:**

**Figure:**
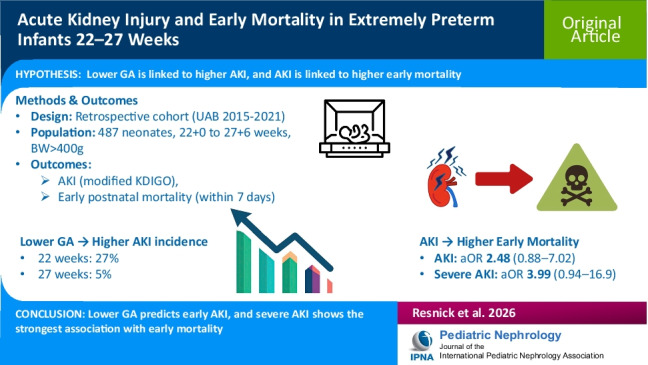
A higher resolution version of the Graphical abstract is available as Supplementary information

**Supplementary Information:**

The online version contains supplementary material available at 10.1007/s00467-026-07214-9.

## Introduction

Extremely low gestational age neonates (ELGANs), defined as neonates born before 28 weeks gestation, remain at high risk for morbidity and mortality despite advances in care. Survival has improved, but these infants still face severe complications such as intraventricular hemorrhage, necrotizing enterocolitis, bronchopulmonary dysplasia, and sepsis [1–3]. Acute kidney injury (AKI) is increasingly recognized as a frequent complication in critically ill neonates, though historically underappreciated. The neonatal kidney is highly vulnerable to ischemic and nephrotoxic injury, and its limited reserve makes preterm infants particularly susceptible to early kidney dysfunction and later chronic kidney disease (CKD) [4–7]. Across age groups, AKI is linked to adverse outcomes, including higher mortality, prolonged hospitalization, and CKD. Neonatal studies likewise demonstrate that AKI is independently associated with increased morbidity and mortality, underscoring the need to define its incidence, risk factors, and impact in high-risk populations such as ELGANs [8–10].

Despite the growing recognition of neonatal AKI, significant gaps remain in our understanding of its epidemiology and significance among ELGANs. Most prior studies of AKI in preterm infants have included broader gestational age ranges, often grouping infants born at 22–23 weeks together with those at later gestations. As a result, data specific to the most immature neonates remain limited. The landscape for these extremely premature neonates is also rapidly evolving, as advances in neonatal care have led to the active treatment and increasing survival of infants born at the lowest gestational ages. This shifting threshold of viability raises new questions regarding the incidence and clinical implications of AKI in these extremely high-risk neonates. In addition, the definition of AKI in neonates remains imperfect. The currently applied criteria, adapted from adult and pediatric KDIGO guidelines, may not fully capture the unique physiology of the developing kidney, especially in ELGANs. Uncertainty around appropriate thresholds and staging limits requires additional observational data on current AKI definitions and critical clinical outcomes [11, 12].

To better understand the relationship between AKI and mortality in extreme prematurity, we performed a retrospective study to address the following aims. The first aim was to evaluate the relationship between gestational age and the incidence of AKI in extremely low gestational age neonates born between 22 and 27 + 6 weeks of gestation. The second aim was to assess whether AKI is independently associated with mortality in the first postnatal week. By clarifying these relationships, we aimed to gain a deeper understanding of the incidence of early AKI and its early prognostic significance in the most premature neonates.

## Methods

### Population

We conducted a retrospective cohort study of all inborn extremely preterm neonates who were actively treated at the University of Alabama at Birmingham (UAB) between January 1, 2015, and December 31, 2021. Neonates were eligible if they were born between 22 + 0 and 27 + 6 weeks gestation, had a birth weight ≥ 400 g, and did not have major congenital anomalies.

To meet the study criteria, neonates were required to have at least two serum creatinine values obtained between postnatal days 3 and 7 for AKI assessment (this threshold was chosen to balance diagnostic precision with sample size, because requiring three or more measurements would have excluded a substantial portion of the cohort); we excluded serum creatinine values from the first 2 days of life to avoid confounding by maternal creatinine, as has been done in previous studies [13]. The data from this study were initially published in the Golden Week Project [14].

This study was approved by the University of Alabama at Birmingham Institutional Review Board (IRB-150908001).

### Definitions

Acute kidney injury (AKI) was defined using the neonatal KDIGO definition [7], based solely on serum creatinine values. This definition was chosen because the urine output data we have is only for 24-h intervals. A rise in creatinine to 1.5–1.9 times the baseline and/or an increase of 0.3 mg/dL or more was classified as stage 1 AKI. A rise to ≥ 2.0 times baseline and/or an absolute creatinine ≥ 2.5 mg/dL was classified as severe AKI (stages 2 or 3). Stages 2 and 3 were combined and are referred to as severe AKI because of the small number of events in these stages. Because urine output was available only as 24-h averages, it did not meet the temporal resolution required for KDIGO staging and therefore could not be incorporated into the AKI definition.

Mortality was defined as death occurring before postnatal day 8 of life. Gestational age (GA) was determined using the best obstetric estimate, based on early prenatal ultrasound and maternal menstrual dating. GA was rounded down to the closest whole week for grouping in descriptive tables and figures. For regression analyses, GA was modeled as a continuous variable.

Covariates included were: study year (for changes in practice over time), race (Black, White, Hispanic, or other; the other category included Native American, Asian, and declined to report), sex, multiple gestation, antenatal steroid exposure, mode of delivery (vaginal, breech, cesarean), birth weight z-score (calculated using Fenton 2013 growth curves), and Apgar score at 5 min. In addition to the primary covariates selected a priori for adjusted modeling, we evaluated several first-week clinical exposures, including vasopressor use, exposure to potentially nephrotoxic medications (vancomycin and aminoglycosides), and necrotizing enterocolitis, as markers of illness severity. These variables were examined descriptively and in sensitivity analyses but were not included in the primary adjusted models due to concerns regarding overadjustment and model instability, given the limited number of early deaths.

### Statistical analysis

Descriptive statistics were used to summarize the cohort's baseline characteristics. Continuous variables were reported as medians with interquartile ranges (IQR), as their distributions were non-normal. Categorical variables were summarized as counts and percentages. Differences between groups were assessed using the Wilcoxon rank-sum test for continuous variables and the χ^2^ or Fisher's exact test for categorical variables, as appropriate.

The Mann–Whitney U test was used to evaluate the association between gestational age and AKI incidence, and the χ^2^ test was used to assess the relationship between AKI and death in the first postnatal week. Gestational age was modeled as a continuous variable. Any AKI was modeled as a binary variable (any AKI vs. No AKI). Severe AKI was modeled as a binary variable (severe AKI vs. no or stage 1 AKI), and the stage of AKI was modeled as a three-level categorical variable (No AKI, stage 1 AKI, stage 2–3 AKI).

Multivariable logistic regression models were adjusted for gestational age, birth weight z-score, 5-min Apgar score, and antenatal steroid exposure. These variables were chosen as they were both found to be significantly different in the population between those who did and did not have AKI or death in the first postnatal week, and are also classically recognized as risk factors for neonatal morbidity and mortality [1]. The study year was added as our group had significant practice changes across these years.

Crude and adjusted odds ratios (ORs) and 95% confidence intervals (CIs) were reported. All statistical analyses were performed using JMP Pro, version 19, and SAS software, version 9.4 (SAS Institute Inc., Cary, North Carolina). A two-sided p-value < 0.05 was considered statistically significant.

## Results

### Population

Of 813 eligible neonates, 326 (40.1%) had < 2 SCr values and were excluded from analysis; thus, 487 (59.9%) had ≥ 2 values and were included in the study. Neonates with ≥ 2 SCr measurements were more likely to be in later years of the study (*p* < 0.0001); no other variables were statistically significantly different, but there was a trend toward lower gestational age among patients with two or more SCr measurements (Supplemental Table 1).

For the included population, the median GA was 25.4 weeks, and the median BW was 700 g. 51% percent of infants were female. Race distribution included 59% Black, 36% White, 4% Hispanic, and 6 infants (1%) classified as other, which included Native American, Asian, or declined to report. 86% received antenatal steroids, and 61% were delivered by cesarean section. Overall, 39 out of 487 patients died in the first postnatal week (8%).

Among 487 neonates included in the AKI analysis (Table 1), 60 (12.3%) developed AKI. 31 had stage 1 and 29 had stage 2–3 AKI (severe AKI). Neonates who developed AKI had significantly lower gestational age, 24 (IQR 23.2–26.0) vs. 25.6 (IQR 24–26.9) weeks (*p* < 0.001). The use of antenatal steroids was lower in the AKI group (44/60 (73.3%) vs. those who did not have steroids (376/427 (88.1%); (*p* < 0.01). There were more AKI events in the later years of the study (*p* = 0.01).

**Table 1:** Baseline characteristics of extremely preterm neonates with and without acute kidney injury (AKI) during the first postnatal week

Characteristic	No AKI (*n* = 427)	Any AKI (*n* = 60)	All (*N* = 487)	*p* value
Study year				**0.01**
2015	65 (15.2)	6 (10.0)	71 (14.6)	
2016	63 (14.8)	4 (6.7)	67 (13.8)	
2017	69 (16.2)	5 (8.3)	74 (15.2)	
2018	62 (14.5)	6 (10.0)	68 (14.0)	
2019	52 (12.2)	10 (16.7)	62 (12.7)	
2020	51 (11.9)	15 (25.0)	66 (13.6)	
2021	65 (15.2)	14 (23.3)	79 (16.2)	
Race				0.14
Other	5 (1.2)	1 (1.7)	6 (1.2)	
Black	245 (57.4)	43 (71.7)	288 (59.1)	
Hispanic	19 (4.5)	1 (1.7)	20 (4.1)	
White	158 (37.0)	15 (25.0)	173 (35.5)	
Sex				0.77
Female	219 (51.3)	32 (53.3)	251 (51.5)	
Male	208 (48.7)	28 (46.7)	236 (48.5)	
Multiparity				0.44
No	330 (77.3)	49 (81.7)	379 (77.8)	
Yes	97 (22.7)	11 (18.3)	108 (22.2)	
Antenatal steroids				** < 0.01**
Missing	1 (0.2)	0 (0.0)	1 (0.2)	
No	50 (11.7)	16 (26.7)	66 (13.6)	
Yes	376 (88.1)	44 (73.3)	420 (86.2)	
Mode of delivery				0.24
Missing	0 (0.0)	1 (1.7)	1 (0.2)	
Breech	23 (5.4)	4 (6.7)	27 (5.5)	
Cesarean	266 (62.3)	30 (50.0)	296 (60.8)	
Vaginal	138 (32.3)	25 (41.7)	163 (33.5)	
Gestational age, wk	25.6 (24.0–26.9)	24.0 (23.2–26.0)	25.4 (23.9–26.6)	** < 0.001**
Birth weight z-score	−0.1 (−0.7 to 0.4)	−0.3 (−1.2 to 0.4)	−0.2 (−0.8 to 0.4)	0.17
Apgar score at 5 min	6 (4–7)	6 (3–7)	6 (4–7)	0.06
Pressors*	73 (17.10)	19 (31.67)	92 (18.89)	**0.01**
Aminoglycosides*	418 (97.89)	60 (100.00)	478 (98.15)	0.61
Vancomycin*	140 (32.79)	17 (28.33)	157 (32.24)	0.56
NEC*	9 (2.11)	1 (1.67)	10 (2.05)	1.00
Death < 8 days				0.11
No	396 (92.7)	52 (86.7)	448 (92.0)	
Yes	31 (7.3)	8 (13.3)	39 (8.0)	

AKI, acute kidney injury. Categorical variables are *n* (% column)

*Variables with *n* (% row)

Continuous variables are reported as the median (IQR)

### AKI incidence and AKI by gestational age

Among the 487 neonates included in the AKI analysis (Table 2), 39 (8.0%) died within the first postnatal week. Several key demographic and perinatal variables differed significantly between survivors and non-survivors. Neonates who died had significantly lower gestational age (*p* < 0.001), lower birth weight z-scores (*p* < 0.0001), and lower 5-min Apgar scores (*p* < 0.001). A later study year was associated with a lower odds of death (*p* < 0.001).

**Table 2:** Baseline characteristics of extremely preterm neonates by early mortality status (death < 8 days)

Characteristic	Survivors (*n* = 448)	Deaths < 8 d (*n* = 39)	All (*N* = 487)	*p* value
Study year				** < 0.001**
2015	63 (14.1)	8 (20.5)	71 (14.6)	
2016	54 (12.1)	13 (33.3)	67 (13.8)	
2017	67 (15.0)	7 (17.9)	74 (15.2)	
2018	63 (14.1)	5 (12.8)	68 (14.0)	
2019	61 (13.6)	1 (2.6)	62 (12.7)	
2020	62 (13.8)	4 (10.3)	66 (13.6)	
2021	78 (17.4)	1 (2.6)	79 (16.2)	
Race				0.32
Other	5 (1.1)	1 (2.6)	6 (1.2)	
Black	261 (58.3)	27 (69.2)	288 (59.1)	
Hispanic	19 (4.2)	1 (2.6)	20 (4.1)	
White	163 (36.4)	10 (25.6)	173 (35.5)	
Sex				0.17
Female	235 (52.5)	16 (41.0)	251 (51.5)	
Male	213 (47.5)	23 (59.0)	236 (48.5)	
Multiparity				0.79
No	348 (77.7)	31 (79.5)	379 (77.8)	
Yes	100 (22.3)	8 (20.5)	108 (22.2)	
Antenatal steroids				0.26
Missing	1 (0.2)	0 (0.0)	1 (0.2)	
No	63 (14.1)	3 (7.7)	66 (13.6)	
Yes	384 (85.7)	36 (92.3)	420 (86.2)	
Mode of delivery				0.43
Missing	1 (0.2)	0 (0.0)	1 (0.2)	
Breech	24 (5.4)	3 (7.7)	27 (5.5)	
Cesarean	276 (61.6)	20 (51.3)	296 (60.8)	
Vaginal	147 (32.8)	16 (41.0)	163 (33.5)	
Gestational age, wk	25.6 (24.0–26.7)	24.0 (23.1–25.3)	25.4 (23.9–26.6)	** < 0.001**
Birth weight z-score	−0.1 (−0.7 to 0.4)	−1.0 (−2.0 to 0.0)	−0.2 (−0.8 to 0.4)	** < 0.0001**
Apgar score at 5 min	6 (4–7)	4 (3–6)	6 (4–7)	** < 0.001**
Pressors*	60 (13.39)	32 (82.05)	92 (18.89)	** < 0.0001**
Aminoglycosides*	439 (97.99)	39 (100.00)	478 (98.15)	1.00
Vancomycin*	139 (31.03)	18 (46.15)	157 (32.24)	0.053
NEC*	9 (1.85)	1 (2.56)	10 (2.05)	0.57

Categorical variables are *n* (% column). *Variables with *n* (% row)

Continuous variables are reported as the median (IQR)

**Fig. 1:**
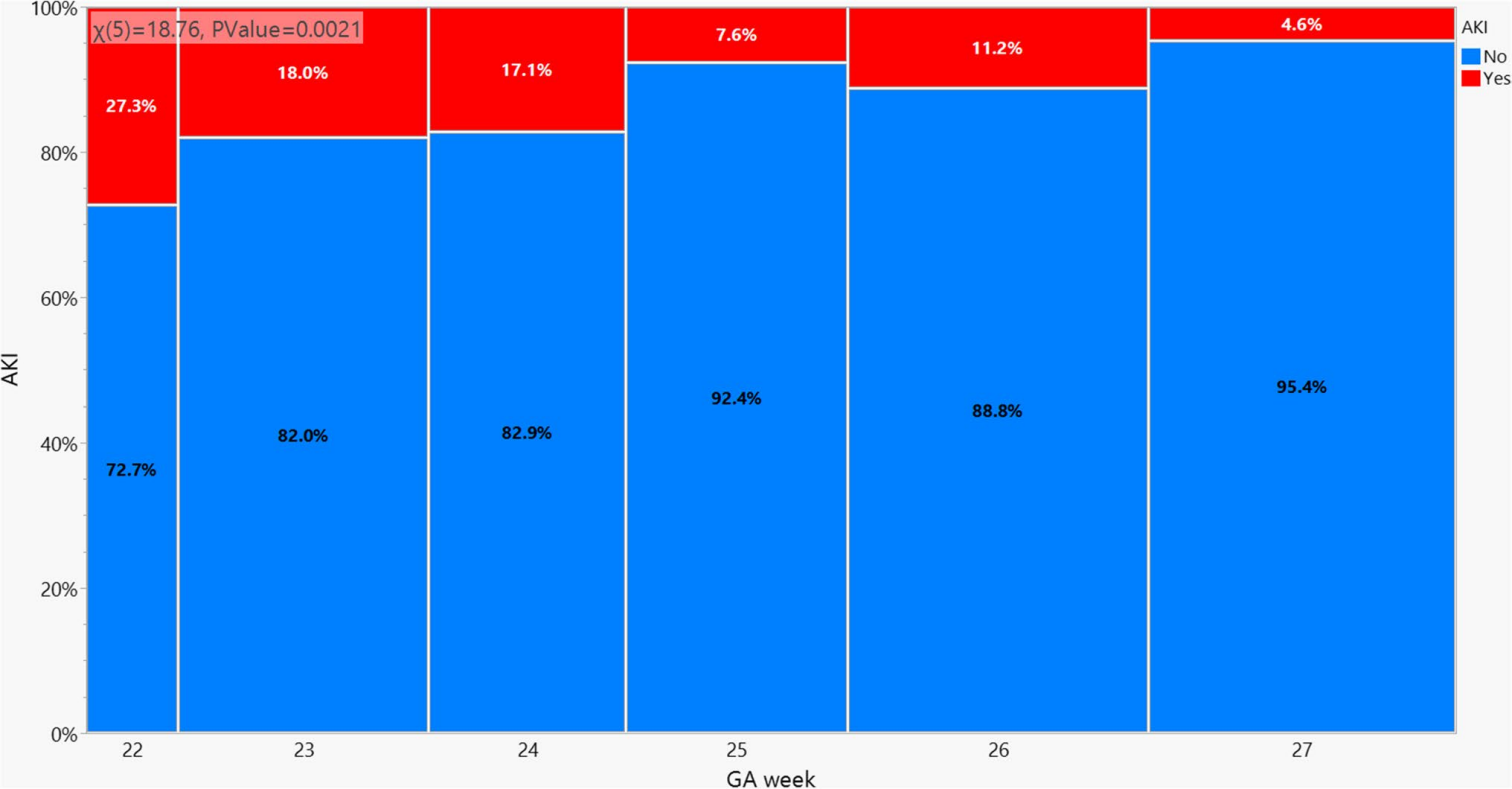
Incidence of AKI by gestational age

**Fig. 2:**
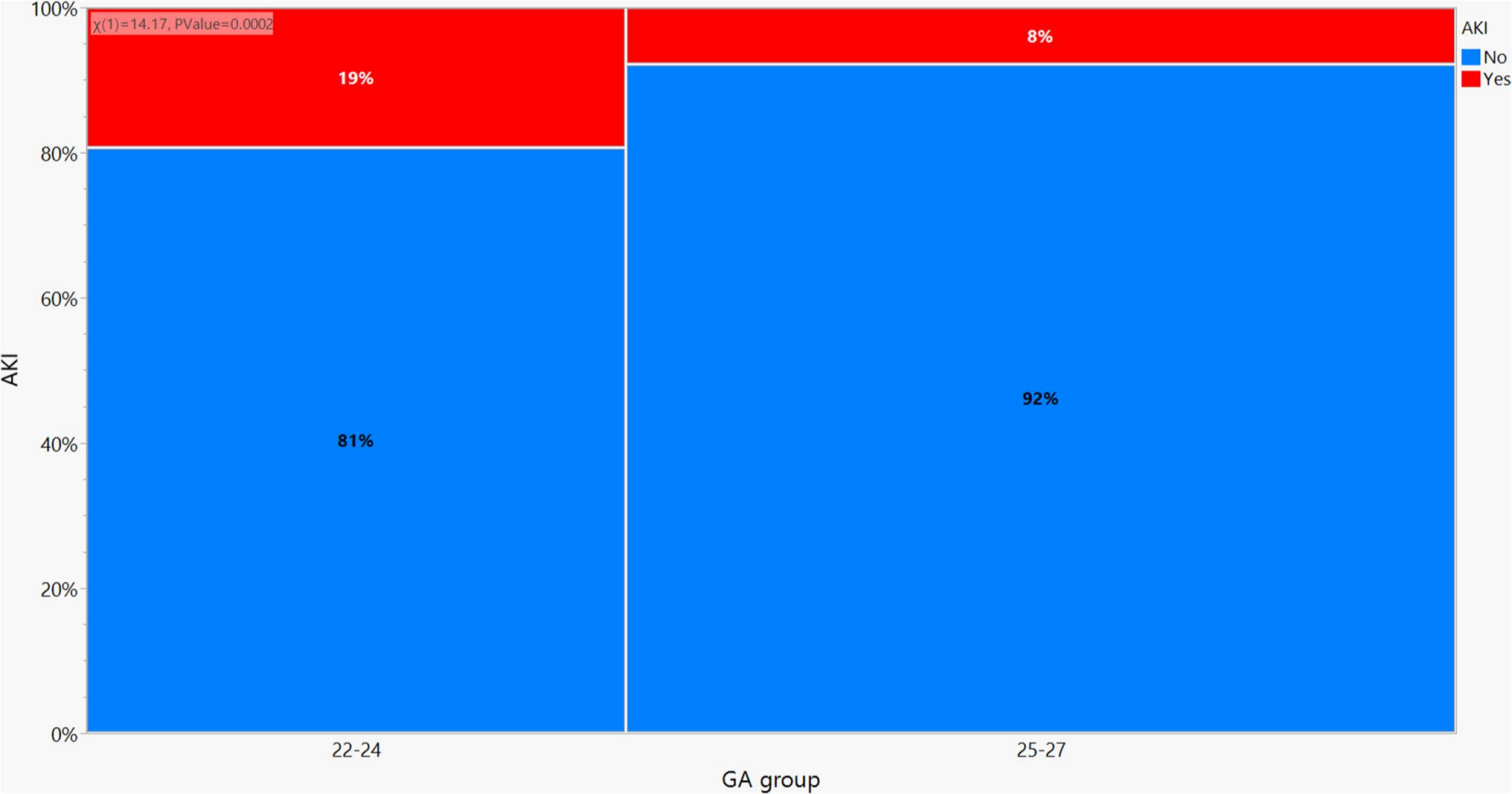
Incidence of AKI by GA groups (22–24 vs. 25–27 weeks)

The incidence of acute kidney injury (AKI) during the first postnatal week varied significantly by gestational age. AKI was most frequent among neonates born at 22 weeks (27.3%) and declined steadily with advancing gestation, reaching 4.6% at 27 weeks (chi-squared test for trend, *p* < 0.01) (Fig. 1). When gestational age was grouped into < 25 weeks vs. ≥ 25 weeks, this pattern persisted: neonates born at 22–24 weeks had a markedly higher incidence of AKI (19.3%) than those born at 25–27 weeks (7.8%; *p* < 0.001) (Fig. 2).

### AKI association with mortality

Table 3 shows the crude and adjusted odds ratios for mortality by different AKI groups. For any AKI vs. No AKI, the odds of death were cOR 1.97 (95% CI 0.81–4.32). After adjustment for gestational age, birth weight z-score, 5-min Apgar score, and antenatal steroid exposure (Model 1), the aOR was 1.40 (95% CI 0.52–3.48). With the addition of study year (Model 2), the aOR was 2.48 (95% CI 0.88–7.02).

**Table 3:** Association of acute kidney injury (AKI) with death within the first 7 days of life

AKI status	cOR (95% CI)	aOR^1^ (95% CI)	aOR^2^ (95% CI)
Any AKI vs. No AKI	1.97 (0.81–4.32)	1.40 (0.52–3.48)	2.48 (0.88–7.02)
Stage 1 vs. No AKI	1.71 (0.49–4.68)	1.50 (0.39–4.69)	1.48 (0.39–5.57)
Severe AKI vs. AKI 0–1	1.93 (0.64–5.87)	1.19 (0.29–3.86)	3.99 (0.94–16.90)

For stage 1 AKI vs. No AKI, the odds of death were cOR 1.71 (95% CI 0.49–4.68). In Model 1, the aOR was 1.50 (95% CI 0.39–4.69), and in Model 2, the aOR was 1.48 (95% CI 0.39–5.57).

For severe AKI vs. AKI stage 0–1, the odds of death were cOR 1.93 (95% CI 0.64–5.87). In Model 1, the aOR was 1.19 (95% CI 0.29–3.86), and in Model 2, the aOR was 3.99 (95% CI 0.94–16.90).

Sensitivity analyses incorporating first-week vasopressor use, nephrotoxin exposure, and necrotizing enterocolitis were performed to assess the robustness of the observed associations; inclusion of these variables did not materially change the direction or magnitude of the AKI–mortality relationships.

**Fig. 3:**
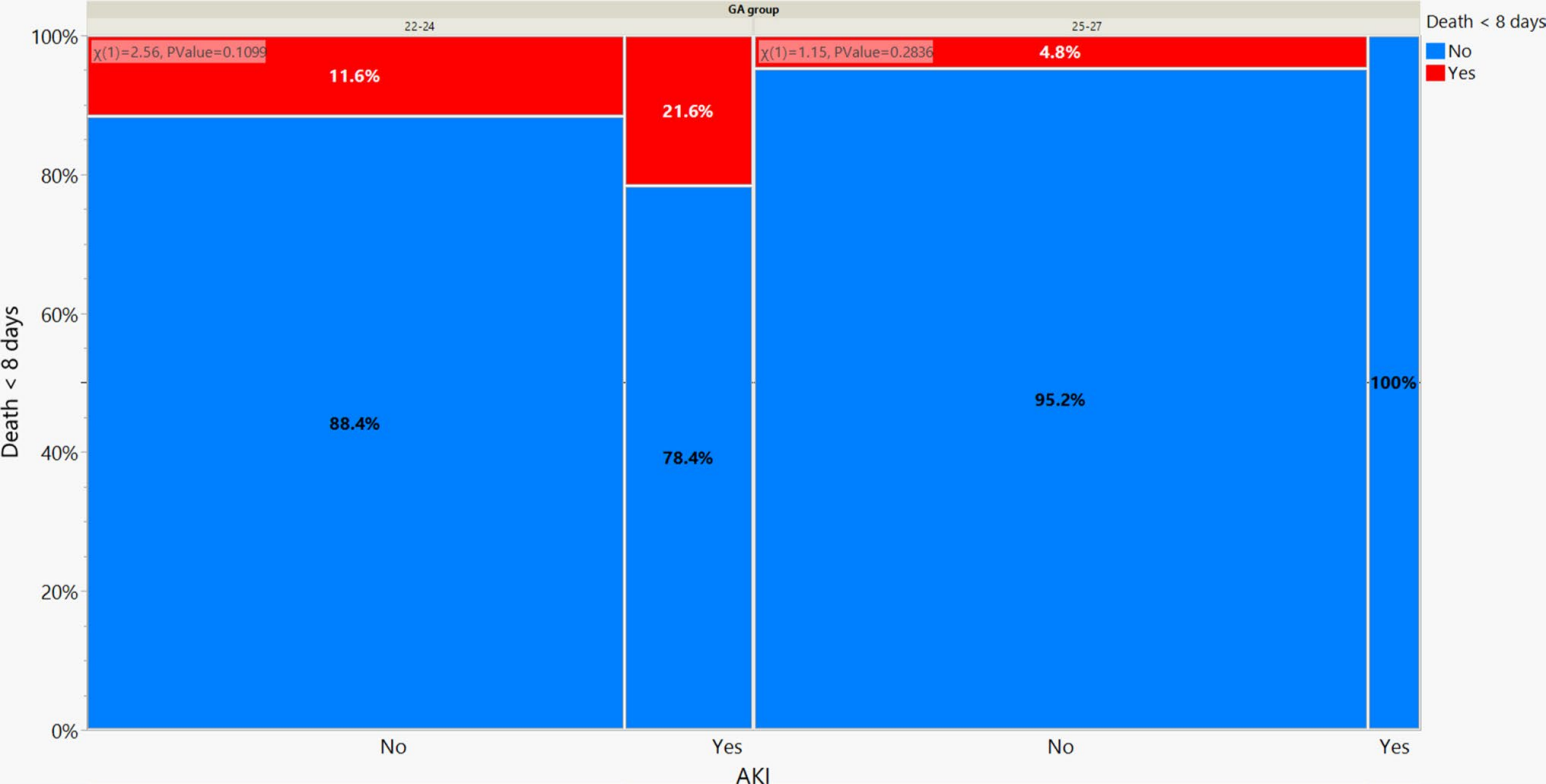
Early mortality by AKI status and gestational age group

In the 22–24 week group, mortality was 21.6% in those with AKI vs. 11.6% without (χ^2^(1) = 2.56, *p* = 0.11). In the 25–27 week group, mortality with AKI was 0% vs. 4.8% without (χ^2^(1) = 1.15, *p* = 0.28) (Fig. 3).

## Discussion

In this cohort of extremely low gestational age neonates, the incidence of AKI was inversely related to gestational age, with the most immature neonates experiencing the highest rates. AKI showed a consistent trend toward higher odds of early mortality, although the associations did not reach statistical significance after adjustment for clinical factors. Notably, severe AKI appeared to carry a stronger signal for mortality compared with milder forms.

In our cohort, 12.3% of ELGANs developed AKI during the first postnatal week, with a striking inverse relationship between gestational age and AKI incidence. AKI occurred in 27% of patients at 22 weeks, compared to 5% at 27 weeks. This distinct gradient emphasizes the profound immaturity of kidney physiology at the lowest gestational ages and highlights the susceptibility of these neonates to early hemodynamic and nephrotoxic stress.

When AKI occurred, it was often severe: almost half of the cases were stage 2–3. This distribution suggests that in ELGANs, kidney injury may not be a subtle finding but rather a manifestation of systemic instability, such as sepsis or cardiovascular compromise. The high proportion of severe cases distinguishes this cohort from some prior reports and underscores the clinical importance of early detection.

We also observed a consistent, though not statistically significant, association between AKI and early mortality. Given the limited number of deaths and the resulting wide confidence intervals, these estimates should be interpreted with caution. Any AKI was associated with approximately double the odds of death, and severe AKI approached a four-fold increase in odds. Wide confidence intervals reflect limited deaths, but directionality suggests AKI contributes to early mortality risk. Notably, the stronger signal for severe AKI relative to stage 1 reinforces its prognostic relevance.

Gestational age stratified analyses revealed an intriguing pattern: among the most immature neonates (22–24 weeks), AKI was associated with higher mortality, whereas no deaths occurred among those with AKI at 25–27 weeks. This lower observed mortality among infants with AKI in the 25–27 week group deviates from prior literature and should not be interpreted as suggesting a protective association. The most likely explanation is the very small number of AKI events and deaths in this subgroup, which makes the estimates unstable. Several additional factors may contribute. Older extremely preterm infants may die from acute respiratory, neurologic, or cardiovascular causes that are less directly related to kidney dysfunction. Ascertainment bias may also play a role if clinicians were more likely to obtain creatinine measurements in infants perceived to have a greater chance of survival, which would selectively underdetect AKI in the sickest infants. Survivorship bias is relevant because infants who die before creatinine measurements on days 3–7 cannot meet AKI criteria. Taken together, these issues suggest that the observed pattern reflects small sample size and methodological limitations rather than a biologic difference in the relationship between AKI and mortality. We also observed temporal changes in practice patterns over the study period. Serum creatinine was obtained more frequently in later years, which likely reflects growing recognition of the importance of kidney function monitoring in ELGANs. This increased surveillance may have contributed to a higher observed incidence of AKI through heightened detection, while simultaneously coinciding with lower mortality rates over time, potentially reflecting broader improvements in care and more proactive management of kidney injury. Additionally, antenatal steroid exposure was associated with a decreased risk of AKI, as has been previously described by Üstün et al. [15]. While the mechanisms underlying this association are not fully understood, steroids may confer kidney protection through improved maturation of kidney and systemic physiology, reduced hemodynamic instability, or mitigation of inflammatory injury [16].

Neonatal AKI has been repeatedly documented in extremely preterm infants, with risk rising as gestational age falls. In a 2024 meta-analysis by Meena et al. examining nearly 100,000 neonates, the incidence of AKI was 30% and severe AKI 15%, and the adjusted odds of mortality were 3.4 (2.9–4.0); however, neonates were not grouped by GA [8]. In a 2022 meta-analysis by Wu et al. including more than 10,000 subjects, the AKI rate was 25%, and the odds of mortality for AKI were (OR = 7.13; 95% CI 5.91–8.60) as well as (OR = 6.30; 95% CI 4.71–8.44; *p* < 0.01) for the extremely low birth weight subgroup, respectively [17]. In the seminal multi-center AWAKEN cohort, AKI occurred in 47.9% of infants born ≥ 22 to < 29 weeks and was independently associated with mortality in this GA stratum (adjusted OR 3.7, 95% CI 1.4–9.7) [10]. This study, however, included a total of 283 subjects in this age group, and a small minority (not specified) had a GA of less than 24 weeks. An important difference in our study was the use of a very narrow time definition of early AKI. This adds precision but also leads to a total decrease in the measured incidence of AKI.

In the PENUT trial, which evaluated ELGANS 24 + 0—27 + 6, 12.2% of subjects had early AKI (days 3–7) (using a similar restriction of the AKI assessment window to serum creatinine values obtained after the first two postnatal days), with 2.2% having stage 2–3 early AKI [18]. This AKI prevalence rate is almost the same as in the current study, although in the current study, nearly half of all AKI events were stage 2 or 3 (29/60, 48.3%). In addition, the PENUT trial enrolled equal numbers of subjects in each of the four GA weeks, whereas this study enrolled all admitted patients, including neonates < 24 weeks. A secondary analysis of this trial showed a trend towards higher mortality in early severe AKI, but this was not statistically significant [9]. Aziz et al. studied neonates under 29 weeks and/or 1,000 g and found an AKI incidence of 44% (primarily based on Scr criterion, 88%) [19]. However, creatinine measurements from the first 2 days of life were not excluded in this study.

This study has several notable strengths. This is one of the largest single-center ELGAN cohorts, providing robust estimates of AKI incidence across a narrow gestational age spectrum. The data were collected in a relatively recent era (2015–2021), ensuring relevance to contemporary neonatal care. All neonates were managed at a tertiary referral center with consistent protocols, thereby enhancing internal validity. We used a standardized neonatal KDIGO definition to ensure comparability and focused on early postnatal AKI (days 3–7), excluding the first two days to reduce maternal creatinine confounding. This approach was used in previous neonatal AKI studies, such as the PENUT study [13]. Finally, the ability to stratify incidence by single weeks of gestation is a unique feature that captures important risk gradients within the ELGAN population.

Several limitations must also be acknowledged. The retrospective design introduces potential bias related to missing data and unmeasured confounders. As a single-center study, generalizability may be limited due to variations in practices and patient populations across institutions. Because the parent Golden Week cohort did not enroll infants weighing less than 400 g, this population could not be included in our analysis, which limits the applicability of our findings to the smallest and most critically ill neonates. We fully recognize that outcomes among infants under 400 g are of significant clinical interest, and future studies that specifically enroll and characterize these neonates will be essential to understanding AKI risk and early morbidity in this group. The temporal relationship between AKI and early mortality cannot be determined in this study. Because the AKI assessment window (days 3 to 7) overlaps with the period when deaths occurred, AKI could have developed as part of the dying process rather than preceding it. Serum creatinine measurements used for AKI classification were obtained before death in all infants who died, but the dataset did not allow us to determine the exact temporal proximity of these measurements to the clinical events leading to death. For these reasons, our findings should be interpreted as associations rather than evidence of a causal effect of AKI on early mortality. Early deaths in extremely preterm infants often reflect profound immaturity or nonrenal clinical deterioration, which further limits the extent to which this outcome can be used to infer the biological role of kidney injury. Given these complexities, AKI in this cohort should be interpreted as an associated finding within the broader context of overall clinical severity rather than as evidence of a causal relationship with early mortality. Serum creatinine testing likely varied by illness severity, leading to ascertainment bias and overestimation of AKI in sicker neonates. Creatinine sampling was not performed daily in all infants, and requiring only two creatinine values during days 3 to 7 may allow physiological fluctuations in early neonatal creatinine to contribute to AKI misclassification. We examined the sampling distribution and found that requiring three or more measurements would have excluded more than half of otherwise eligible infants, substantially reducing statistical power and generalizability. We therefore selected a threshold of at least two measurements as a pragmatic balance between diagnostic precision and cohort retention, but acknowledge that sparse sampling remains an important limitation. Furthermore, AKI was defined solely by creatinine measurements, without urine output data, which likely underestimated the true incidence. To address this limitation, we performed a sensitivity analysis evaluating minimum mean daily urine output as a predictor of early mortality. Urine output was not associated with death (*p* = 0.83), and its inclusion did not materially change the estimated association between AKI and mortality. These findings suggest that adding urine output to our definition would not have meaningfully altered the observed associations. Although we evaluated several first-week clinical exposures, including vasoactive medication use, nephrotoxin exposure, and necrotizing enterocolitis, these factors likely reflect downstream manifestations of critical illness and may lie on the causal pathway between extreme prematurity, AKI, and early mortality. Residual confounding by illness severity, infection, fluid balance, and other unmeasured factors, therefore, remains possible. Mortality was defined only within the first postnatal week, and therefore, later associations of early AKI with longer-term mortality or morbidity were not captured. Finally, the few severe AKI cases and deaths limited precision, resulting in wide confidence intervals.

Our findings underscore the need for further investigation into AKI among ELGANs. Future studies should focus on whether refining definitions of neonatal AKI to account for the unique physiology of extremely preterm neonates is needed. Incorporating urine output, emerging biomarkers, and continuous transdermal GFR measurements will better capture the metrics needed to identify kidney disease. Incorporation of other outcomes may improve the accuracy of neonatal AKI diagnosis and staging.

Multi-center, prospective cohorts will be critical for validating these findings across diverse NICU populations and practice environments, including those under 400 g at birth. Such studies should systematically capture important clinical variables, including vasoactive medication use, infection status, and fluid balance, to better delineate risk factors for AKI. In parallel, efforts to standardize creatinine monitoring and urine output practices could reduce ascertainment bias and enhance comparability across centers.

Finally, linking early AKI to longer-term outcomes — including survival beyond the first postnatal week, neurodevelopment, and later kidney health — remains a critical priority [20]. Understanding these trajectories will not only clarify the prognostic significance of AKI in ELGANs but also identify opportunities for targeted interventions to mitigate its impact.

## Conclusions

In this large cohort of extremely low gestational age neonates, the risk of acute kidney injury was highest in the most immature neonates. While the association between AKI and early mortality did not reach statistical significance, our estimates were directionally consistent with prior studies and suggest that AKI may be a key contributor to poor outcomes. These findings support the need for continued kidney monitoring in ELGANs and support ongoing efforts to refine AKI definitions, standardize monitoring practices, and explore preventive strategies to improve outcomes for this fragile population.

## Supplementary Information

Below is the link to the electronic supplementary material.

Graphical abstract (PPTX 199 KB)

Supplementary file1 (DOCX 19 KB)

## Data Availability

The datasets generated and analyzed during the current study are available from the corresponding author on reasonable request.
