# Supplementary material for: Acute kidney injury and early mortality in extremely preterm neonates born at 22–27 weeks gestation
Source: Pediatr Nephrol. 2026 Apr 10;41(9):3023–32. doi: 10.1007/s00467-026-07214-9 (PMC13424248; doi:10.1007/s00467-026-07214-9)
Supplement: Supplementary file 1 — Graphical abstract (PPTX 199 KB) [file 467_2026_7214_MOESM1_ESM.pptx]

## Slide 1
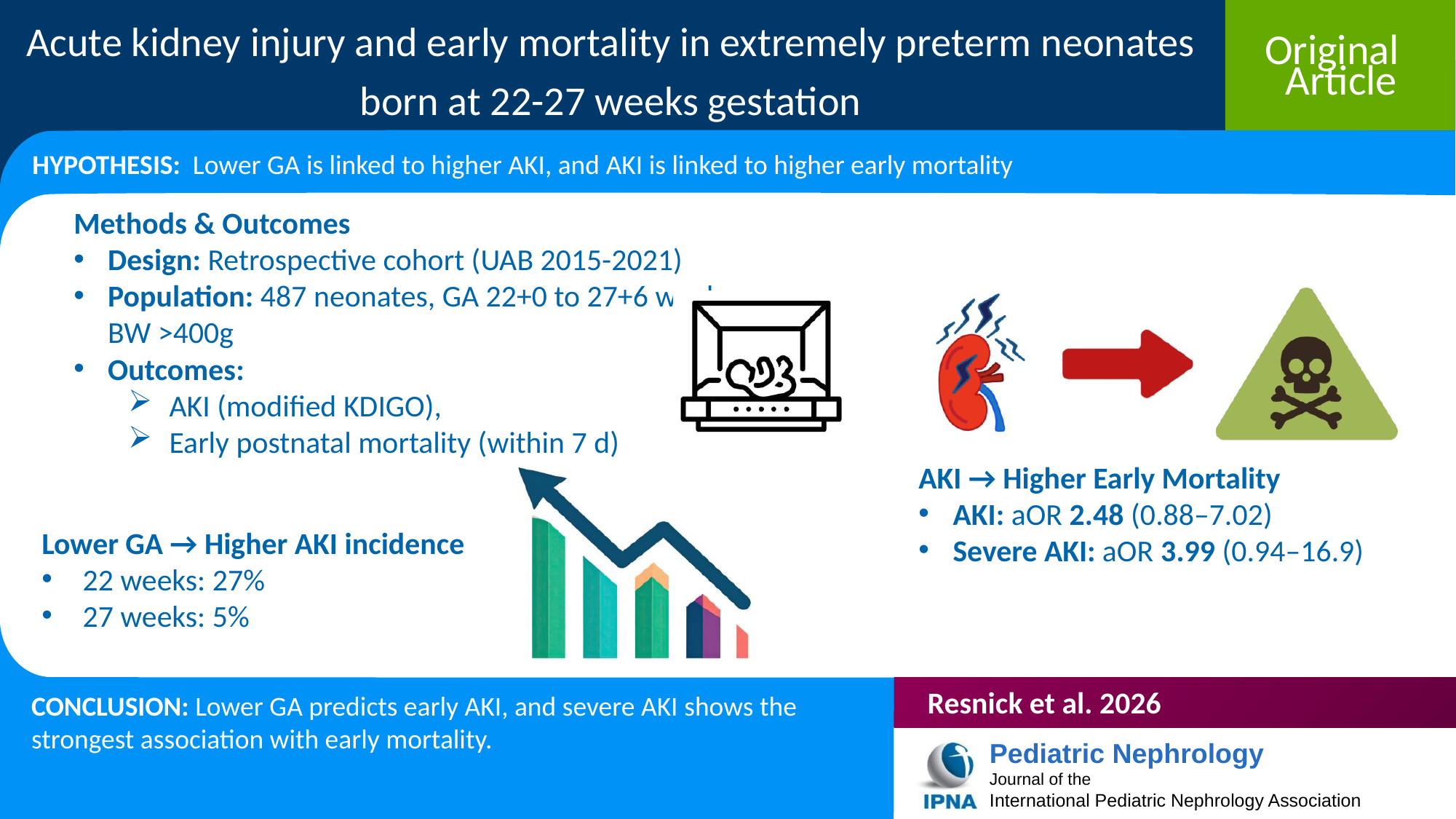

Acute kidney injury and early mortality in extremely preterm neonates born at 22-27 weeks gestation
HYPOTHESIS: Lower GA is linked to higher AKI, and AKI is linked to higher early mortality
Methods & Outcomes
Design: Retrospective cohort (UAB 2015-2021)
Population: 487 neonates, GA 22+0 to 27+6 weeks, BW >400g
Outcomes:
AKI (modified KDIGO),
Early postnatal mortality (within 7 d)
AKI → Higher Early Mortality
AKI: aOR 2.48 (0.88–7.02)
Severe AKI: aOR 3.99 (0.94–16.9)
Lower GA → Higher AKI incidence
22 weeks: 27%
27 weeks: 5%
Resnick et al. 2026
CONCLUSION: Lower GA predicts early AKI, and severe AKI shows the strongest association with early mortality.
