# Supplementary material for: Acute kidney injury and early mortality in extremely preterm neonates born at 22–27 weeks gestation
Source: Pediatr Nephrol. 2026 Apr 10;41(9):3023–32. doi: 10.1007/s00467-026-07214-9 (PMC13424248; doi:10.1007/s00467-026-07214-9)
Supplement: Supplementary file 2 — Supplementary file1 (DOCX 19 KB) [file 467_2026_7214_MOESM2_ESM.docx]

**Supplemental Table 1. Baseline characteristics of extremely preterm infants by availability of ≥2 serum creatinine (SCr) measurements between postnatal days 3-7**

| **Characteristic** | **<2 SCr values (n=326)** | **≥2 SCr values (n=487)** | **All (N=813)** | **p value** |
| --- | --- | --- | --- | --- |
| **Study year** |  |  |  | **<0.001** |
| 2015 | 65 (19.9) | 71 (14.6) | 136 (16.7) |  |
| 2016 | 54 (16.6) | 67 (13.8) | 121 (14.9) |  |
| 2017 | 60 (18.4) | 74 (15.2) | 134 (16.5) |  |
| 2018 | 49 (15.0) | 68 (14.0) | 117 (14.4) |  |
| 2019 | 45 (13.8) | 62 (12.7) | 107 (13.2) |  |
| 2020 | 39 (11.9) | 66 (13.6) | 105 (12.9) |  |
| 2021 | 14 (4.3) | 79 (16.2) | 93 (11.4) |  |
| **Race** |  |  |  | 0.53 |
| Black | 187 (57.3) | 288 (59.1) | 475 (58.4) |  |
| Hispanic | 14 (4.3) | 20 (4.1) | 34 (4.2) |  |
| White | 124 (38.0) | 173 (35.5) | 297 (36.5) |  |
| Other | 1 (0.3) | 6 (1.2) | 7 (0.7) |  |
| **Sex** |  |  |  | 0.87 |
| Female | 170 (52.2) | 251 (51.5) | 421 (51.8) |  |
| Male | 156 (47.8) | 236 (48.5) | 392 (48.2) |  |
| **Multiparity** |  |  |  | 0.78 |
| No | 251 (77.0) | 379 (77.8) | 630 (77.5) |  |
| Yes | 75 (23.0) | 108 (22.2) | 183 (22.5) |  |
| **Antenatal steroids** |  |  |  | 0.59 |
| Missing | 0 (0.0) | 1 (0.2) | 1 (0.1) |  |
| No | 40 (12.3) | 66 (13.6) | 106 (13.0) |  |
| Yes | 286 (87.7) | 420 (86.2) | 706 (86.8) |  |
| **Mode of delivery** |  |  |  | 0.60 |
| Missing | 1 (0.3) | 1 (0.2) | 2 (0.2) |  |
| Breech | 13 (4.0) | 27 (5.5) | 40 (4.9) |  |
| Cesarean | 200 (61.4) | 296 (60.8) | 496 (61.0) |  |
| Vaginal | 112 (34.4) | 163 (33.5) | 275 (33.8) |  |
| **Gestational age, wk** | 26.0 (24.1-27.0) | 25.4 (23.9-26.6) | 25.5 (24.0-26.9) | 0.064 |
| **Birth weight z-score** | -0.1 (-0.8 to 0.5) | -0.2 (-0.8 to 0.4) | -0.1 (-0.8 to 0.4) | 0.29 |
| **5 min Apgar score** | 7 (4-7) | 6 (4-7) | 6 (4-7) | 0.41 |
| **Death <8 days** |  |  |  | 0.19 |
| No | 291 (89.3) | 448 (92.0) | 739 (90.9) |  |
| Yes | 35 (10.7) | 39 (8.0) | 74 (9.1) |  |

Abbreviations: SCr, serum creatinine. Categorical variables are n (% column). Continuous variables are reported as the median (IQR).

Results Summary:
Infants with ≥2 SCr measurements were more likely to be in later years of the study (p < 0.0001); no other variables were statistically significantly different, but there was a trend toward lower gestational age in the patients who had two or more serum creatinine measurements obtained (39/487 (8%) vs. 35/326(10.7%) ; p< 0.064)
